# Molecular docking studies of 3-bromopyruvate and its derivatives to metabolic regulatory enzymes: Implication in designing of novel anticancer therapeutic strategies

**DOI:** 10.1371/journal.pone.0176403

**Published:** 2017-05-02

**Authors:** Saveg Yadav, Shrish Kumar Pandey, Vinay Kumar Singh, Yugal Goel, Ajay Kumar, Sukh Mahendra Singh

**Affiliations:** 1 School of Biotechnology, Institute of Science, Banaras Hindu University, Varanasi, Uttar Pradesh, India; 2 Centre for Bioinformatics, School of Biotechnology, Institute of Science, Banaras Hindu University, Varanasi, Uttar Pradesh, India; 3 Department of Zoology, Institute of Science, Banaras Hindu University, Varanasi, Uttar Pradesh, India; Istituto di Genetica Molecolare, ITALY

## Abstract

Altered metabolism is an emerging hallmark of cancer, as malignant cells display a mammoth up-regulation of enzymes responsible for steering their bioenergetic and biosynthetic machinery. Thus, the recent anticancer therapeutic strategies focus on the targeting of metabolic enzymes, which has led to the identification of specific metabolic inhibitors. One of such inhibitors is 3-bromopyruvate (3-BP), with broad spectrum of anticancer activity due to its ability to inhibit multiple metabolic enzymes. However, the molecular characterization of its binding to the wide spectrum of target enzymes remains largely elusive. Therefore, in the present study we undertook *in silico* investigations to decipher the molecular nature of the docking of 3-BP with key target enzymes of glycolysis and TCA cycle by PatchDock and YASARA docking tools. Additionally, derivatives of 3-BP, dibromopyruvate (DBPA) and propionic acid (PA), with reported biological activity, were also investigated for docking to important target metabolic enzymes of 3-BP, in order to predict their therapeutic efficacy versus that of 3-BP. A comparison of the docking scores with respect to 3-BP indicated that both of these derivatives display a better binding strength to metabolic enzymes. Further, analysis of the drug likeness of 3-BP, DBPA and PA by Lipinski filter, admetSAR and FAF Drug3 indicated that all of these agents showed desirable drug-like criteria. The outcome of this investigation sheds light on the molecular characteristics of the binding of 3-BP and its derivatives with metabolic enzymes and thus may significantly contribute in designing and optimizing therapeutic strategies against cancer by using these agents.

## Introduction

It is well recognized that malignant cells display altered metabolism, which is recognized as an emerging hallmark of cancer, through which the malignant cells support their bioenergetic and biosynthetic machinery [1,2]. The altered metabolism of malignant cells is mainly realized by the up-regulation of enzymes catalyzing glycolysis and to a lesser extent the TCA cycle [3,4]. Thus, recent therapeutic approaches envisage to inhibit the expression and activity of such enzymes which regulate and drive the altered metabolic machinery of the neoplastic cells [5,6]. In this quest, most of the inhibitors of cancer metabolism identified so far are known to specifically inhibit the activity of a single target enzyme [7]. On the contrary the tumor cells possess a tremendous capability to combat such approaches through compensatory adaptive strategies, which can be one of the major limitations of using a single enzyme-specific inhibitor [8,9]. Consequently, it becomes imperative to identify inhibitors capable of simultaneously targeting multiple enzymes of tumor metabolic pathways. One of such upcoming inhibitors is an alkylating agent known as 3-bromopyruvate (3-BP), which has been demonstrated to display a wide spectrum of antineoplastic actions [10–13]. However, the precise mechanisms underlying the antitumor actions are still under extensive investigation. The main mechanism by which 3-BP is understood to exert its antineoplastic action is by hampering ATP generation, which is generally attributed to the wide spectrum of metabolic targets inhibited by 3-BP including: hexokinase 2 (HK 2), 3-phosphoglycerate kinase (PGK), glyceraldehyde 3-phosphate dehydrogenase (GAPDH), lactate dehydrogenase (LDH), pyruvate dehydrogenase complex (PDC), succinate dehydrogenase (SDH), α-ketoglutarate dehydrogenase, isocitrate dehydrogenase (IDH), glyoxalase 1 & 2 and serine hydroxyethyltransferase [10,12–15]. Further, most of these target enzymes of 3-BP are found to be specifically up-regulated in cancer cells [1,2]. Therefore, with such a wide spectrum of enzyme inhibitory potential, 3-BP can usher a complete breakdown of cancer cell metabolism [10,12]. Thus, 3-BP could prove to be a superior chemotherapeutic agent compared to other conventional metabolic inhibitors that target only a single enzyme of a specific metabolic pathway.

In view of the emerging importance of 3-BP as an anticancer agent [10,13,16], attention is being paid to precisely understand the molecular mechanisms of its antitumor actions including the characterization of its binding to target enzymes, which will aid in optimizing its therapeutic applications. Our survey of literature indicated that there is no report so far to define the molecular nature of the binding of 3-BP to various target enzymes. Further, despite the availability of 3-BP derivatives DBPA and PA, with demonstrated biological actions like modulation of fatty acid level, immunosuppressive actions, insulin sensitivity, anti-proliferative activity and anticholinesterase activity [17–20], their potential for binding to target enzymes of metabolic pathways remains unexplored. Considering the utility of recent advances in the field of bioinformatics and *in silico* analytical tools to characterize molecular interactions, the present study was carried out to decipher the biochemical nature of the binding of 3-BP and its derivatives to important target enzymes of glycolysis and TCA cycle. The investigation also analyzed the drug likeness potential of 3-BP and its derivatives.

## Materials & methods

**Fig 1:**
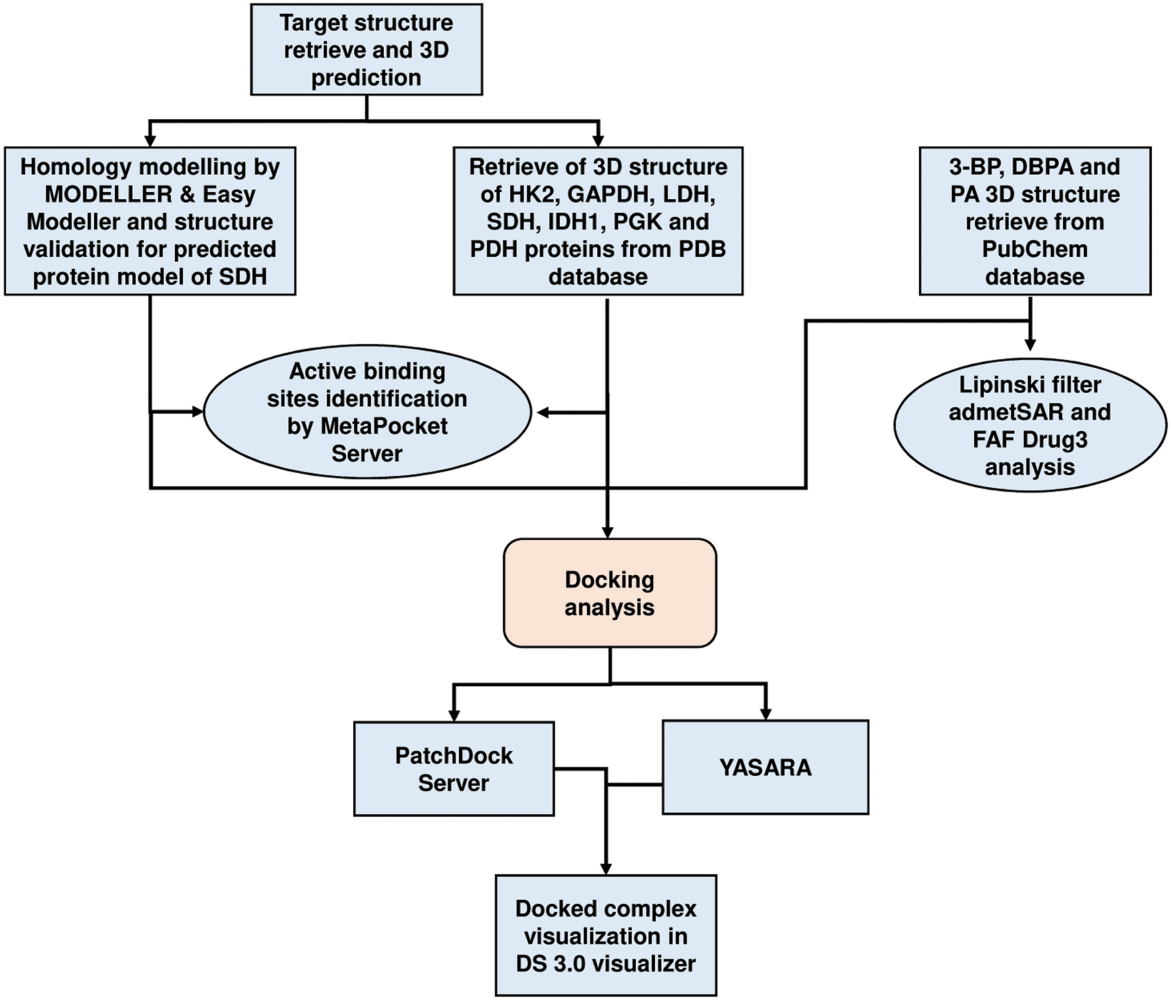
Flowchart depicting schematic strategy of *in silico* analysis.

This investigation included retrieval of the 3D structure of target enzymes and ligands from PDB and PubChem databases, respectively. The 3D structure of SDH was predicted by homology modelling and validated thorough RAMPAGE and PDBSum server. Active binding sites were identified by MetaPocket server. Docking was performed by PatchDock server and YASARA tool, whereas docking complexes were visualized by Discovery Studio 3.0. The drug likeness was analysed through Lipinski filter, admetSAR and FAFDrug3. A flow chart of the methodology is depicted in Fig 1.

### Retrieval of target enzyme structures

Protein Data Bank (http://www.rcsb.org/pdb/home/home.do) was used for retrieving the structure of the following enzymes of glycolysis and TCA cycle, of *Homo sapiens* origin, which are recognized as targets of 3-BP: LDH (1I0Z, DOI: 10.2210/pdb1i0z/pdb), GAPDH (1U8F, DOI: 10.2210/pdb1u8f/pdb), HK 2 (2NZT, DOI: 10.2210/pdb2nzt/pdb), IDH 1 (4UMX, DOI: 10.2210/pdb4umx/pdb), PGK (4AXX, DOI: 10.2210/pdb4axx/pdb) and PDH (3EXE, DOI: 10.2210/pdb3exe/pdb)[21]. The criteria for selection of the indicated structures were based on PDB advance BLAST analysis and the structures use in this study were those displaying maximum score and query cover in BLAST.

### Homology modelling of human SDH

The crystal structure for human SDH was unavailable in the PDB databank, thus the strategy of homology modelling was utilized to generate its 3D structure. The Human SDH protein sequence (NP_004159.2) was retrieved from the NCBI database for structure prediction. Templates were selected using homology search from NCBI and PDB databases. Three templates (3AEF; 2H88; 1YQ3) were used for homology modelling using EasyModeller 4.0 and MODELLER. The homology of these templates with SDH is shown in S1 Table. The homology of the selected templates with SDH was above 89% with respect to percent identity and 95% for percent positivity. These tools are used to generate automated three-dimensional structures of proteins based on homology modelling [22,23]. Based on the available templates, it was possible to model 614 amino acids length from Ile^51^ to Tyr^664^, hence, in our model, amino acid residue Ile^51^ is indicated as residue one. The predicted SDH model was validated using RAMPAGE and PDBsum server [24,25]. The SDH quality estimation was carried out by SAVES, ERRAT [26] and VADAR servers [27]. Resolution of the modelled SDH structure was calculated using ResRrox server [28]. The generated model of SDH was visualized using Discovery Studio 3.0 [29].

### Ligand retrieval

The structures 3-BP (CID:70684) [30] and its derivatives DBPA (CID:120293) and PA (CID:71374591) were retrieved from the PubChem database [10,17,19,31,32]. These structures were used for docking calculation. The selected 3D structure of the ligands was retrieved from PubChem Compound database in SDF format followed by conversion in the PDB format and optimization using Discovery Studio. Further shape complementarity principle was applied with clustering RMSD 4.0 for docking calculations.

### Prominent binding site prediction

Prior to docking analysis, prominent binding site prediction of LDH, GAPDH, HK 2, SDH, PDH, PGK and IDH 1 were carried out by MetaPocket 2.0 server [33]. Top 3 major binding pockets were retrieved for analysis of active binding residues and comparison of the docking results.

### Docking analysis

PatchDock server, a geometry based molecular docking algorithm[34], was used for docking analysis of 3-BP and its derivatives to the indicated target enzymes. The PDB files of ligand and target enzymes were uploaded to PatchDock server for docking analysis, using cluster RMSD at default value of 4.0 and protein-small ligand complex type as the analysis parameters. Analysis on PatchDock yielded results for geometric shape complementarity score (GSC score) and approximate interface area (AI area). Additional docking tool YASARA (Yet Another Scientific Artificial Reality Application), an AutoDock based tool for molecular docking and virtual screening was used for analysing dissociation constant (Kd) and binding energy of the docked complexes [35,36].

### Analysis of drug likeness of 3-BP, DBPA and PA

The drug likeness prediction of 3-BP, DBPA and PA was carried out by Lipinski filter (http://www.scfbio-iitd.res.in/software/drugdesign/lipinski.jsp), according to which an orally active drug should comply to a minimum of four of the five laid down criteria for drug likeness namely: molecular mass, cLogP, hydrogen donor and acceptor and molar refractive index [37]. Moreover, the properties of ligand with respect to prediction of adsorption, distribution, metabolism, excretion and toxicity (ADMET) were analysed by admetSAR (http://lmmd.ecust.edu.cn:8000/), which is reported as an useful tool in drug discovery [38]. This tool was utilized for predicting important descriptors of drug likeness. FAF-Drug 3 (http://fafdrugs3.mti.univ-paris-diderot.fr/) was used to predict additional ADMET properties of 3-BP and its derivatives. This tool assists in filtering studies for selection of good drug candidates for drug development projects [39]. The SDF (Structure Data Format) file of the 3-BP and its derivatives were downloaded from PubChem Database to calculate ADME properties using default parameters.

## Results and discussion

### Determination of the target enzyme molecular structure

**Fig 2:**
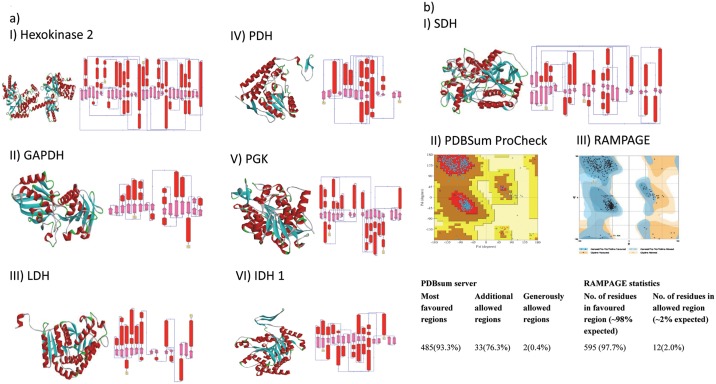
Structural details of target enzymes. (a) Molecular structures of the indicated human target enzymes of 3-BP, except SDH, were retrieved from PDB data bank. Figure shows 3D ribbon structures along with topology of secondary structures. (b) The structure of SDH (i) was predicted by homology modelling as described in materials and methods, the quality of predicted SDH model was estimated by RMPAGE (ii) and PDBSum server (iii).

The structural details of target enzymes (LDH, GAPDH, HK 2, PDH, PGK and IDH 1) were obtained from PDB data bank, whereas the structure of SDH was determined by homology modelling approach as the same was unavailable at PDB data bank. The molecular structural details of these enzymes, except SDH, are presented in Fig 2a. Molecular details pertaining to the target enzymes obtained from PDBSum server, included topology of the secondary structure, β and γ turns, parameter of helical geometry, β bulge and hairpin, disulphide bridge and H-bond, psi loops and helix interactions (Table 1). Information on these parameters aids in determining the overall structural organization of proteins, their structural motifs and also in predicting and analyzing the docking pockets of proteins susceptible for convenient binding of the ligands. A comparison of various target enzymes on these parameters revealed an overall similar organizational pattern, displaying flexible areas of the proteins vulnerable for docking with ligands [40].

**Table 1:** Details of structural elements of the target enzymes.

Proteins	Sheets	Beta alpha beta units	Beta hairpins	Beta bulges	Strands	Helices	Helix-helix interacs	Beta turns	Gamma turns
**HK2**	6	2	10	5	26	39	43	71	6
**GAPDH**	2	2	4	3	16	13	6	41	6
**LDH**	4	4	4	2	13	16	14	22	5
**SDH**	7	1	7	8	24	23	19	52	9
**PDH**	4	3	2	1	12	15	20	31	4
**PGK**	4	6	2	1	17	23	19	25	6
**IDH 1**	3	2	3	3	14	19	19	18	3

### Homology modelling for predicting 3-D structure of SDH

Homology modelling approach was used for predicting the tree dimensional structure of SDH, which is based on predetermined homologous templates [41]. By using three selected templates (3AEF; 2H88; 1YQ3) homology modelling of SDH was carried out as described in materials and methods. The generated model of SDH (Fig 2b) displayed DOPE score-70735.20, which is a reliable statistical potential to assess quality of homology models in protein structure prediction [41]. The generated SDH model was further validated with RAMPAGE, PDBSum, SAVES, VADAR and ResProx servers. RAMPAGE analysis of the predicted SDH model showed 97.7% residues in favored region, which is close to the requisite percentage of 98 for validating a model (Fig 2b). Favoured regions of Ramachandran plot are energetically and sterically stable conformations of residues characterized by values of torsion angles ψ and ϕ. Using PDBsum server, 93.3% residues were observed to be in the most favored regions. On the PDBSum server a good quality model should have more than 90% residues in most favored region. The ERRAT, ProSA, ResProx and QMEAN servers were used for quality checking, which revealed that the overall quality of the predicted SDH model is reliable compared with the template structures 1YQ3, 3AE1 and 2H88 (S1 Table). The hydrogen bonds (H-bonds) statistics for predicted model was calculated and found to be closer with the expected Mean H-bond Distance, Mean H-bond Energy and Residue with H-bonds (S2 Table). The PDB file of the generated SDH model is shown in S1 File.

### Docking analysis of 3-BP

**Fig 3:**
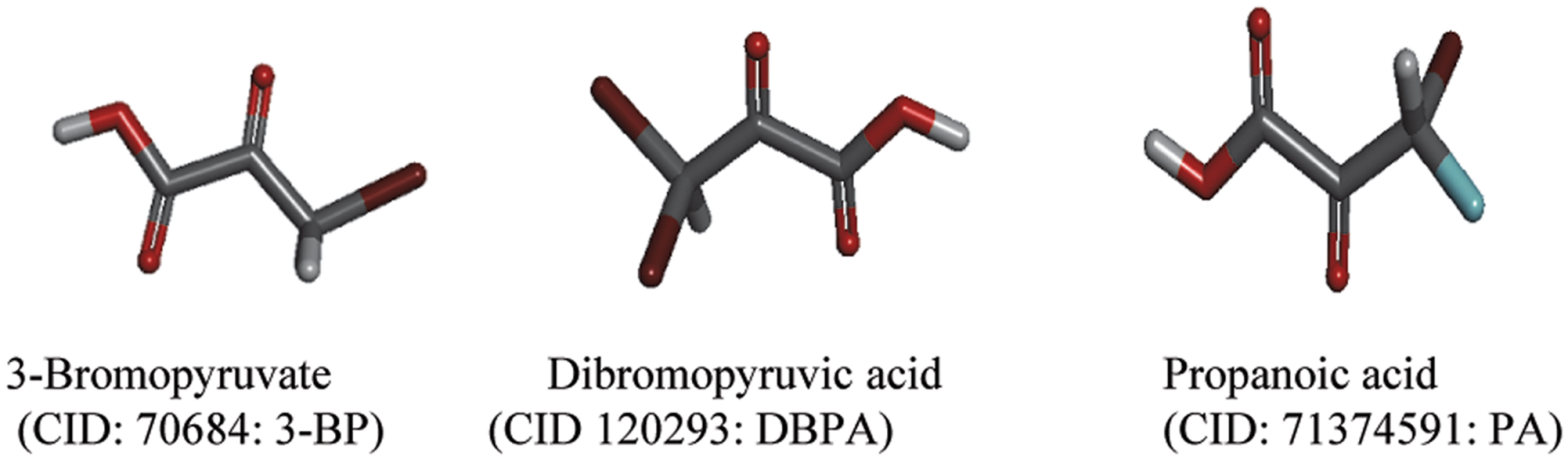
3D structure of retrieved ligands. Structure of 3-BP and its derivatives DBPA and PA were retrieved from PubChem compound database. The PubChem CIDs (Compound Identifier) are shown along with each ligands.

**Fig 4:**
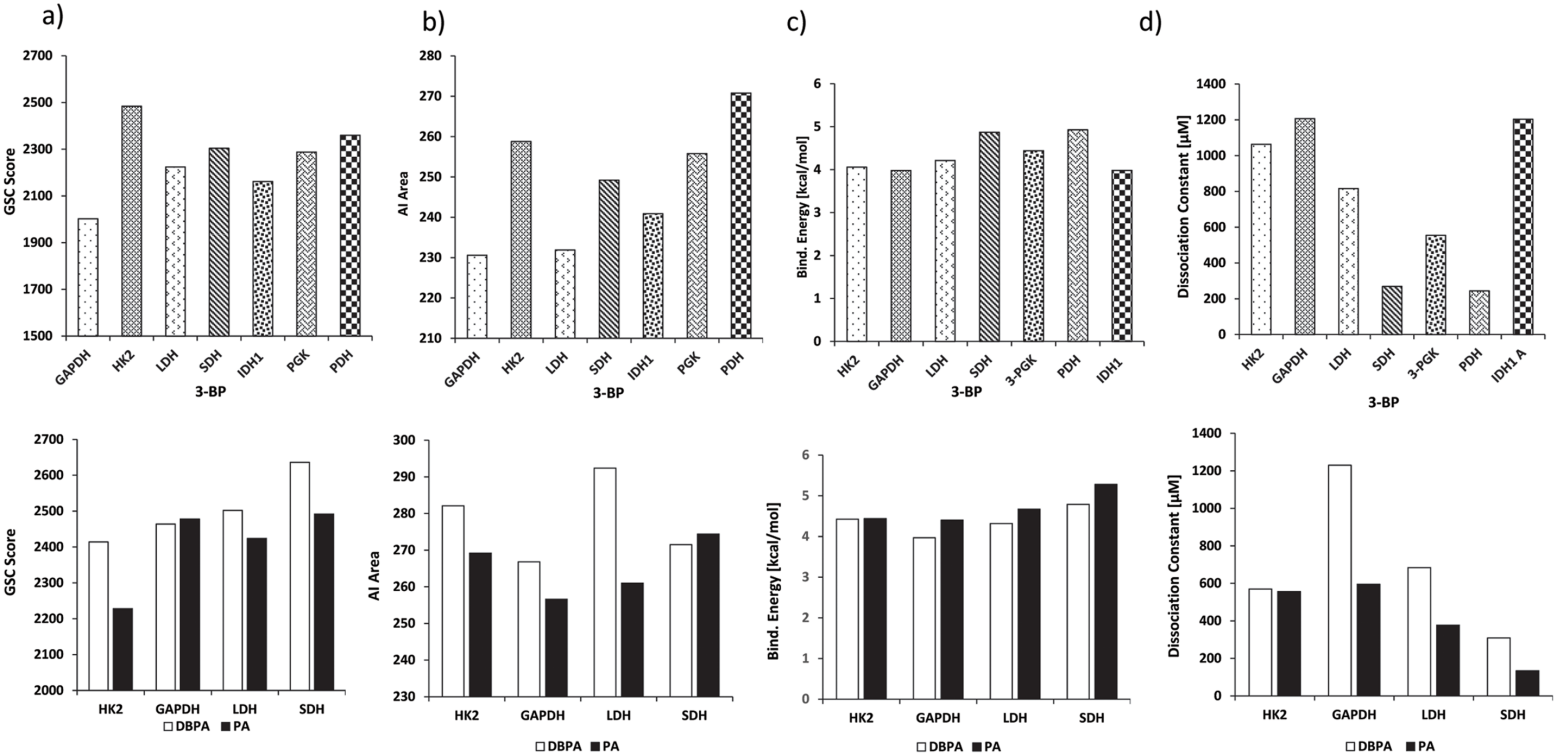
Docking analysis of 3-BP and its derivatives by PatchDock and YASARA. Docking properties analysis between indicated ligands and target enzymes was evaluated on various parameters including GSC score (a), AI area (b) by PatchDock server and binding energy (c) and dissociation constant (Kd) (d) by YASARA.

**Fig 5:**
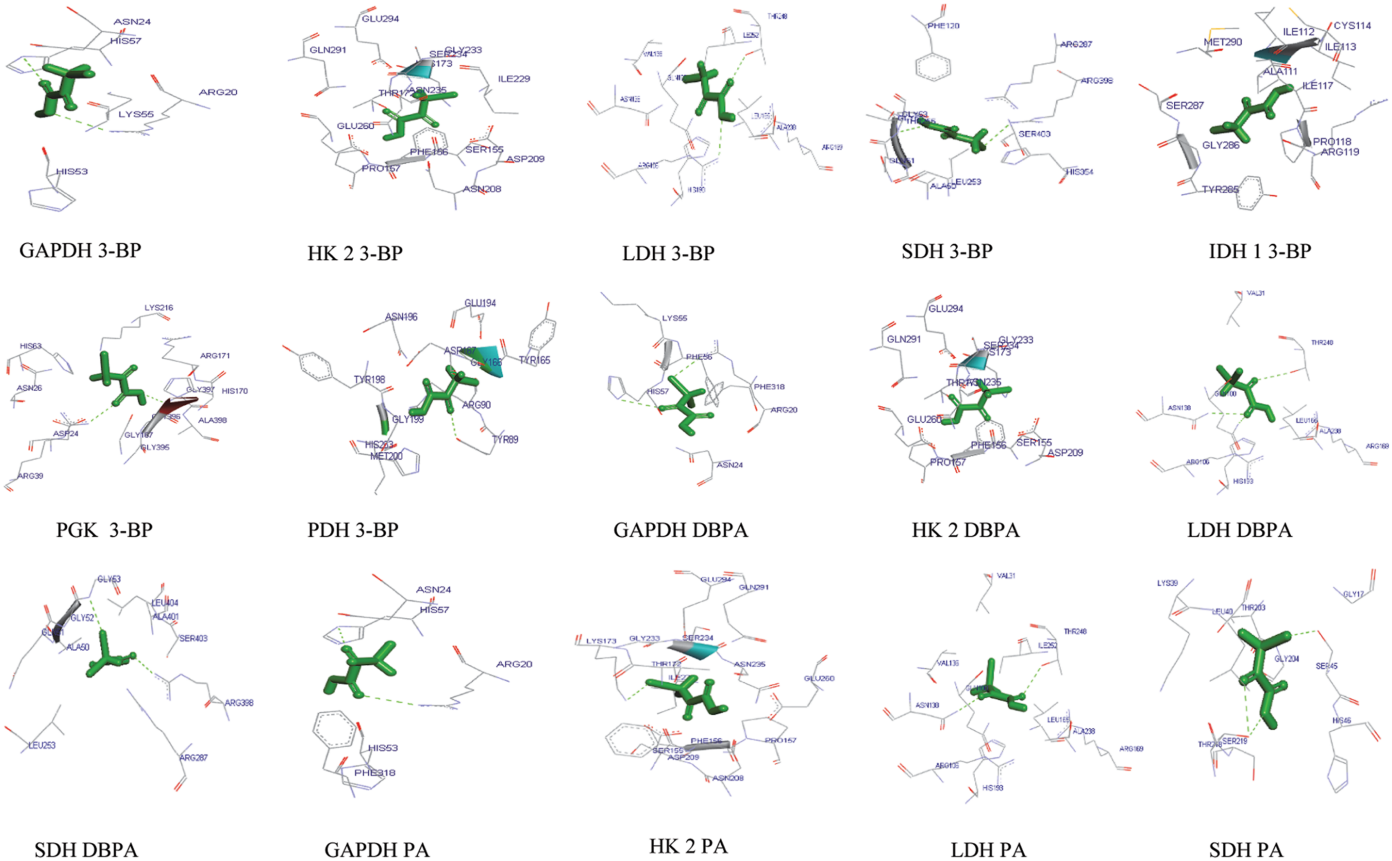
Visualization of docked complexes. Figure shows 3-D models of docked complexes as visualized by Discovery Studio 3.0, showing interactions of 3-BP and its derivatives with the target enzymes.

In order to characterize docking properties of 3-BP with the indicated enzymes, the ligand structure of 3-BP, retrieved from PubChem database was analysed for docking (Fig 3), using PatchDock server and YASARA followed by visualization of the docked complexes by Discovery studio 3.0. The prominent binding sites were also predicted through MetaPocket 2.0 server (S3 Table). Docking of 3-BP with the target enzymes of glycolysis and TCA cycle was studied with respect to following parameters: (a) interacting amino acids (b) ligand and protein atoms involve in hydrogen bonding (Table 2), and (c) GSC score, AI area, and predicted binding energy and Kd (Fig 4). Most of the interacting amino acidic residues, as defined by PatchDock analysis, were found to be those within prominent binding sites as predicted by MetaPocket. As shown in S3 Table, except GAPDH, most of the reported residues of the binding site for the indicated enzymes were common to those predicted by MetaPocket server. The reason for the non-matching of residues for GAPDH could be due to non-availability of X-ray structure of this enzyme with natural substrate or other inhibitors except for NAD. Further, interacting residues common to reported binding site residues, shown in Table 2, also corroborate the commonness between reported binding site residues and interacted residues involved in docking. Docking of 3-BP with most target enzymes, except HK 2 and IDH 1, revealed involvement of H-bonding between 3-BP and amino acids residues located in the prominent binding sites 1, 2 & 3. Hydrogen bonds in association with other non-covalent interactions such as ionic interactions, hydrophobic interactions, van der Waals forces play a role in protein-ligand interactions, however, the H-bonds are not necessarily important for the protein-ligand interactions as the removal of H-bond forming groups has been observed in strengthening the protein-ligand interaction [42] when forcing the system into an unfavorable geometry. 3-BP has been reported to react with thiol groups in target enzymes in a selective manner through second order nucleophilic substitution (S_N_^2^) reaction mechanism [43]. The docking and/or alkylation by 3-BP could trigger conformational alteration of enzymes active sites, leading to a reduction of their catalytic activity. Moreover, specific alkylation of proteins by 3-BP is dependent on biochemical properties of target amino acids to create nucleophilic site [43]. Alkylation-dependent inhibition of target enzymes by 3-BP could mostly implicate modification of substrate binding active site [15,44,45] In case of enzymes with lower dissociation constants namely SDH and PGK, the H-bonding involved Gly^52^ and Arg^398^ and Arg^39^ and Gly^397^ whereas for PDH it was through Tyr^89^. An overall analysis of amino acids involved in docking revealed the implication of Arg, Asn. Gly, His, Ser and Thr as most common amino acids at the prominent docking sites, signifying their role in determining the binding strength and relative contribution in formation of H-bonds. Further, all the aforementioned amino acid residues found in the binding pockets may also be involved in the creation of a local environment, which aids in recognition and orientation of 3-BP and its derivatives. Indeed, the critical role of amino acids in docking has already been demonstrated, in correct docking orientation [46]. Charge and hydrophobicity of amino acids also plays an important role in docking interactions [47]. Further, docking could also be impacted by surrounding pH conditions [48] Moreover, these amino acids with Pro and Ala can collectively contribute to formation of hydrophobic interactions, van der Walls forces, ionic bonds, charged interactions, and H-bonds. The binding strength of the docked complexes was analyzed through GSC score, AI area and predicted binding energy **(**Fig 4) showed good docking strength to all the target enzymes, with the GSC score being comparable for most enzymes, however higher for HK 2. These observations were corroborated by AI area with 3-BP showing larger AI area for HK 2, SDH and PGK. Similarly, the predicted binding energy for all target enzymes was in range of 3.981 to 4.927, indicating strong docking of the drug. YASARA based calculations of dissociation constant (Kd) were found to be within 1207.50 to 269.30 μM, considered indicative of a stable binding. Based on these parameters HK 2 followed by PDH and SDH qualify as preferred target. Further, the docked complexes were visualized by Discovery Studio 3.0. (Fig 5) to display various interactions involved in the ligand-target docking. Based on these parameters (Fig 4) HK 2 was most preferred target enzyme followed by PDH and SDH. Although, 3-BP has been reported to inhibit all the target enzymes shown in this study, to the best of our knowledge, no study has experimentally determined K_i_ and K_d_ of 3-BP with target enzymes used in this study.

**Table 2:** Docking calculations depicting interacting residues, binding site residues and atoms involved in H-bonding along with interacting residues common to reported active binding site residues.

ligand	Protein Name	Interacted residues	Site No. and binding site residues	Ligand and protein atom involved in H-bonding	Interacting residues common with reported active binding sites
3-BP	GAPDH	Arg^20^, Asn^24^, His^53^, Lys^55^, His^57^	Binding site2:Arg^20^, Asn^24^, His^53^, Lys^55^, His^57^	O2; Arg^20^:NH1 O4; His ^57^:ND1	none
	HK 2	Ser^155^, Phe^156^, Pro^157^, Lys^173^, Asp^209^, Gly^233^, Ser^234^, Asn^235^, Glu^260^, Glu^294^	Binding site2: Ser^155^, Lys^173^, Asp^209^, Binding site3: Phe^156^, Gly^233^, Ser^234^, Asn^235^, Glu^260^, Glu^294^	No H-bonding	Ser^155^, Phe^156^, Pro^157^, Lys^173^, Asp^209^, Ser^234^, Asn^235^, Glu^260^, Glu^294^
	LDH	Arg^106^, Asn^138^, Leu^165^, His^193^, Ala^238^, Thr^248^, Ile^252^	Binding site1:Arg^106^, Asn^138^, His^193^, Ala^238^ Binding site2:Leu^165^, Thr^248^, Ile^252^	O2; Arg^106^:NH2 O4; Thr^248^:OG1	Arg^106^, Asn^138^, Leu^165^, His^193^, Ala^238^, Thr^248^, Ile^252^
	SDH	Gln^51^, Gly^52^, Thr^255^, Arg^287^, His^354^, Arg^398^	Binding site1: Gln^51^, Gly^52^, Thr^255^, Arg^287^, His^354^, Arg^398^ Binding site2: His^354^	O2; Gly^52^:N BR1; Arg^398^:NH2	
	PGK	Asn^26^, Arg^39^, Gly^167^, His^170^, Lys^216^, Gly^396^, Gly^397^, Ala^398^	Binding site 1: Asn^26^, Gly^167^, His^170^, Lys^216^, Gly^396^, Gly^397^ Binding site 2: Arg^39^	O4; Arg^39^:ND2 O2; Gly^397^:N	Asn^26^, Arg^39^, Gly^167^, Lys^216^, Gly^396^, Gly^397^,
	PDH	Tyr^89^, Gly^166^, Asp^167^, Asn^196^, Tyr^198^, Gly^199^, Met^200^	Binding site 1: Tyr^89^, Gly^166^, Asp^167^, Asn^196^, Tyr^198^, Gly^199^, Met^200^	O3;Tyr^89^:OH	Tyr^89^, Asp^167^, Asn^196^, Tyr^198^, Gly^199^
	IDH1	Ala^111^, Ile^112^, Ile^113^, Pro^118^, Tyr^285^, Gly^286^, Ser^287^	Binding site 2: Ala^111^, Ile^112^, Ile^113^, Pro^118^, Tyr^285^, Gly^286^, Ser^287^	No H-bonding	none
DBPA	GAPDH	Arg^20^,Phe^56^,His^57^	Binding site2:Arg^20^,Phe^56^, His^57^	BR1; Arg^20^:NH1 O3; His^57^:ND1	none
	HK 2	Ser^155^,Phe^156^,Pro^157^, Thr^172^,Lys^173^,Gly^233^, Asn^235^,Glu^260^,Gln^291^,Glu^294^	Binding site2:Ser^155^, Thr^172^, Lys^173^, Gln^291^ Binding site3:Phe^156^, Pro^157^,Gly^233^, Asn^235^, Glu^260^,Glu^294^	No H-bonding	Ser^155^,Phe^156^, Pro^157^, Thr^172^, Lys^173^, Asn^235^, Glu^260^,Gln^291^, Glu^294^
	LDH	Gln^100^,Arg^106^,Asn^138^, Ala^238^,Thr^248^	Binding site1:Gln^100^, Arg^106^, Asn^138^, Ala^238^ Binding site2:Thr^248^	O5; Gln^100^:NE2 O5; Asn^138^:ND2 O3; Thr^248^:OG1	Gln^100^,Arg^106^, Asn^138^, Ala^238^, Thr^248^
	SDH	Ala^50^,Gly^52^,Gly^53^, Leu^253^,Arg^287^,Arg^398^, Ser^403^	Binding site1:Ala^50^,Gly^52^, Gly^53^, Leu^253^,Arg^287^, Arg^398^, Ser^403^	BR2; Gly^53^:N O5; Arg^398^:NH2	
PA	GAPDH	Arg^20^,Asn^24^,His^53^, His^57^	Binding site2:Arg^20^,Asn^24^, His^53^,His^57^	O5; Arg^20^:NH1	none
	HK 2	Ser^155^,Phe^156^,Pro^157^, Lys^173^,Asn^208^,Asp^209^,Gly^233^,Ser^234^,Asn^235^, Glu^260^	Binding site2:Ser^155^, Lys^173^, Asp^209^ Binding site3:Phe^156^, Pro^157^, Asn^208^, Gly^233^, Ser^234^,Asn^235^, Glu^260^	BR1; Lys^173^:NZ	Ser^155^,Phe^156^, Pro^157^, Lys^173^, Asn^208^, Asp^209^, Ser^234^,Asn^235^, Glu^260^
	LDH	Val^136^,Asn^138^, Ala^238^,Thr^248^,Ile^252^	Binding site1:Val^136^, Asn^138^, Ala^238^ Binding site2: Thr^248^, Ile^252^	O4; Asn^138^:ND2 O5; Thr^248^:OG1	Val^136^,Asn^138^, Ala^238^,Thr^248^, Ile^252^
	SDH	Ser^45^,His^46^,Thr^203^, Thr^218^, Ser^219^	Binding site1: Ser^45^,His^46^,Thr^203^, Thr^218^, Ser^219^ Binding site2: Ser^45^,His^46^,Thr^203^,Thr^218^, Ser^219^	BR1; Ser^45^:OG O3; Thr^218^:OG1 O4; Thr^218^:OG1	

### Docking of important targets by 3-BP derivatives DBPA and PA

In the next section of this study we analysed the docking of 3-BP derivatives DBPA and PA with indicated target enzymes, through which 3-BP is best known to inhibit tumor cell metabolism, namely HK 2, GAPDH, LDH and SDH. DBPA showed high GSC score and AI area accompanied by a lower dissociation constant compared to scores obtained with the docking of 3-BP to the respective enzymes (Fig 4), indicating a better docking potential of DBPA to the target enzymes. Dissociation constant of PA was also comparatively lower than DBPA, whereas GSC score and AI area were somewhat overlapping to that of 3-BP with the respective target enzymes. DBPA showed binding to the interacting amino acids of prominent binding pocket of site 2 for GAPDH, HK 2, LDH and site 1 for SDH, whereas PA showed interacting amino acids in prominent bonding pocket 2 for GAPDH, HK 2 and site 1 for LDH and SDH. In both cases no H-bond were involved in docking with HK 2 (Table 2). The docking of DBPA and PA with GAPDH, HK 2, LDH and SDH as visualized by Discovery Studio is shown in Fig 5 displaying prominent interactions**.**

### Testing of drug likeness of 3-BP, DBPA and PA

The drug likeness of 3-BP and its derivatives was analysed by Lipinski filter and the properties of the ligands with respect to prediction of adsorption, distribution, metabolism, excretion and toxicity by admetSAR. Both of these tools are highly useful in predicting drug likeness and drug designing [38]. All the three drugs, on the shown parameters, displayed values indicating their potential of being used as drugs for application in biological systems. cLogP of 3-BP was -5.730900 while that for DBPA and PA -9.349401 and -0.195100 respectively, indicating that all the three agents qualified to be used as drugs. cLogP values less than zero are considered favorable for drug likeness of a given compound as it is correlated to water solubility and hence contributes to bioavailability [49]. On all other parameters of Lipinski filter and admetSAR, the score of the three drugs were comparable (Table 3a and 3b). The results generated for the ADME/tox properties of 3-BP and its derivatives DBPA and PA using FAFDrugs 3, ADME/tox filtering, are listed in Table 4. Findings were in agreement with Lipinski’s rule of five and passed through the filtering analysis.

**Table 3:** The drug likeness of 3-BP and its derivatives.

**a) Lipinski filter analysis**			
	**Name of agent**		
**Lipinski filters**	**3-BP**	**DBPA**	**PA**
**Mass**	167.0	247.0	185.0
**Hydrogen bond donor**	0	0	0
**Hydrogen bond acceptors**	3	3	3
**cLogP**	-5.730900	-9.349401	-0.195100
**Molar Refractivity**	16.327499	16.034500	19.702499
**b) admetSAR analysis**			
	**Name of agent**		
**Parameters**	**3-BP**	**DBPA**	**PA**
**Absorption**			
**Blood-Brain Barrier**	BBB+	BBB+	BBB+
**Human Intestinal Absorption**	HIA+	HIA+	HIA+
**Caco-2 Permeability**	Caco2-	Caco2-	Caco2-
**P- glycoprotein Substrate**	Non-substrate	Non-substrate	Non-substrate
**AMES Toxicity**	AMES toxic	AMES toxic	AMES toxic
**Carcinogens**	Non carcinogens	carcinogens	carcinogens
**Acute Oral Toxicity**	III	III	III
**Rat Acute Toxicity**	2.7374 LD50, mol/kg	2.1371 LD50, mol/kg	2.4945 LD50, mol/kg
**CYP450 Substrate and Inhibitor**	Non-substrate, Non-inhibitor	Non-substrate, Non-inhibitor	Non-substrate, Non-inhibitor
**hERG**	Weak inhibitor	Weak inhibitor	Weak inhibitor

The drug likeness of 3-BP and its derivatives was analysed by Lipinski filter (a) admetSAR (b).

**Table 4:** FAF Drug3 analysis for ADME/tox properties of 3-BP and its derivatives.

Ligand used	Oral Bioavail. (VEBER & GAN)	Sol. (mg/l)	Rot. Bon.	Rig. Bon.	C	Ratio H/C	No charges	LogP (octanol / water)	tPSA	Status
**3-BP**	Good	59430.21	2	2	3	1.33	1	-1.03	57.2	Accepted
**DBPA**	Good	30628.86	2	2	3	1.67	1	-2.08	57.2	Accepted
**PA**	Good	43518.96	2	2	3	1.67	1	-1.45	57.2	Accepted

FAF Drug3 analysis of drug likeness on indicated parameter.

## Conclusion

**Fig 6:**
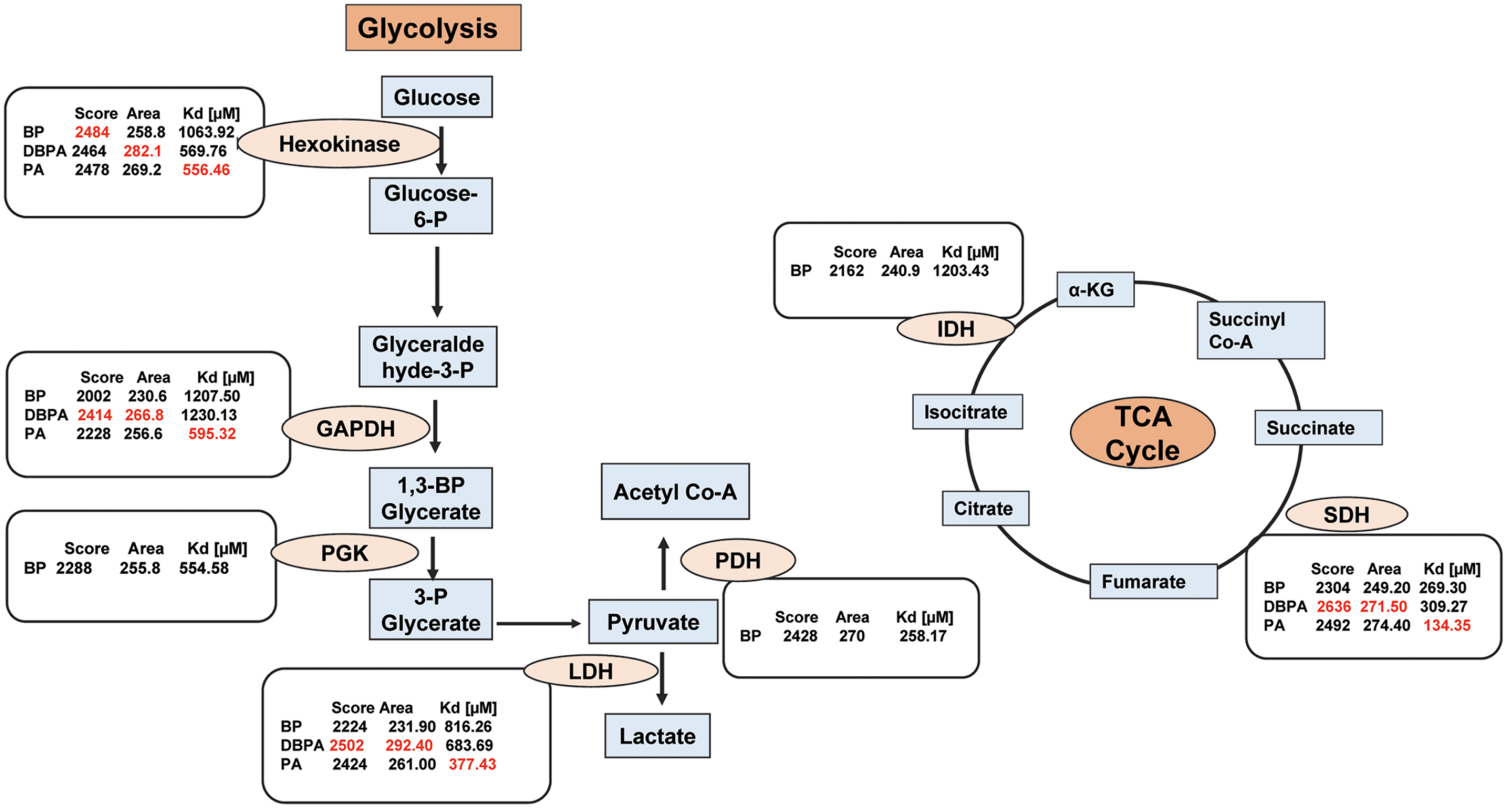
Summary of docking analysis. Figure presents summary of the binding of 3-BP to various target enzymes of glycolysis and TCA cycle, indicating the wide spectrum of its targets.

The present investigation sheds a new light on the potential interactions between 3-BP, DBPA and PA to metabolic enzymes, indicating the involvement of multiple interactions such as H-bonds, hydrophobic interactions and van der Waals forces, depending on the amino acid composition of binding sites and chemical properties of the docking agents and target enzymes (Fig 6). Further, this study is first of its kind to demonstrate the molecular mechanism(s) underlying the docking of chemotherapeutic agent 3-BP to the target enzymes of glycolysis and TCA cycle, indicating the tremendous potential of this agent in catastrophing the bioenergetic machinery of malignant cells. Moreover, derivatives of 3-BP, DBPA and PA were docked with the key target enzymes of glycolysis and TCA cycle. A comparison of the docking scores with respect to 3-BP indicated that both of these derivatives display a better binding strength to metabolic enzymes and thus hold a promising potential to be explored for their antitumor activity. The comparison of docking scores revealed that on average DBPA was superior to 3-BP and PA. However, till date there has been no study to explore the antineoplastic and related pharmacological properties of these derivatives. As these derivatives are also predicted to satisfy drug likeness criteria, they are worth of further investigations in terms of both *in vitro* tests and *in vivo* actions on in tumor bearing hosts to assess and optimize their therapeutic efficacy. The findings of this study are expected to have an impact on the current emergence of research focus on the wide spectrum of the antineoplastic actions of 3-BP by investigating the unexplored aspects of the molecular nature of its interaction with target enzymes. Taken together these observations will usher a new area of research for utilizing the derivatives of 3-BP for novel antineoplastic strategies.

## Supporting information

S1 Table Template details for SDH protein using PDB Advance BLAST. (DOCX)

S2 Table **a)** Structural quality estimation results obtained from ERRAT, ProSA, ResProx, and QMEAN servers. **b)** VADAR: Hydrogen Bonds (H-bonds) statistics for predicted model.
(DOCX)

S3 Table Prominent binding site and their residues identification of predicted (SDH) and retrieved (GAPDH, HK 2, LDH, PDH, PGK and IDH 1) protein models by MetaPocket 2.0 server and reported binding site residues from PDB X-ray crystal structures. (DOCX)

S1 File PDB file of the generated SDH model. (PDB)
