# Supplementary material for: Molecular docking studies of 3-bromopyruvate and its derivatives to metabolic regulatory enzymes: Implication in designing of novel anticancer therapeutic strategies
Source: PLoS One. 2017 May 2;12(5):e0176403. doi: 10.1371/journal.pone.0176403 (PMC5413015; doi:10.1371/journal.pone.0176403)
Supplement: S1 Table — (DOCX) [file pone.0176403.s001.docx]

**S1 Table.** Template details for SDH protein using PDB Advance BLAST

| Target Protein with Accession ID | Organism | Template Used | Organism | Identity (%) | Positives  (%) |
| --- | --- | --- | --- | --- | --- |
| SDH (NP_004159.2) | *Homo sapiens* | 3AE1 | Porcine  (*Sus scrofa*) | 94 | 96 |
|  |  | 2H88 | Avian  (*Gallus gallus*) | 89 | 95 |
|  |  | 1YQ3 | Avian  (*Gallus gallus*) | 89 | 95 |
