# Supplementary material for: Molecular docking studies of 3-bromopyruvate and its derivatives to metabolic regulatory enzymes: Implication in designing of novel anticancer therapeutic strategies
Source: PLoS One. 2017 May 2;12(5):e0176403. doi: 10.1371/journal.pone.0176403 (PMC5413015; doi:10.1371/journal.pone.0176403)
Supplement: S2 Table — a) Structural quality estimation results obtained from ERRAT, ProSA, ResProx, and QMEAN servers. b) VADAR: Hydrogen Bonds (H-bonds) statistics for predicted model. (DOCX) [file pone.0176403.s002.docx]

**S2 Table.**

**a)** Structural quality estimation results obtained from ERRAT, ProSA, ResProx, and QMEAN servers

**b)** VADAR: Hydrogen Bonds (H-bonds) statistics for predicted model

| **a)** | | | |  | | |  |  | |  | |
| --- | --- | --- | --- | --- | --- | --- | --- | --- | --- | --- | --- |
| Target/ Template Proteins | | | | Overall Quality Factor | | | ProSA  Z-score | Predicted resolution(Å) | | QMEAN | |
| SDH | | | | 91.045 | | | -9.44 | 2.045 | | 0.76 | |
| **b)** | | | |  | | |  |  | |  | |
| Receptor Proteins | | Mean H-bond Distance | | | | Mean H-bond Energy | | | Residue with H-bonds | | |
|  |  | | Observed | | Expected | Observed | | Expected | Observed | | Expected |
| SDH | | 2.2(sd=0.3) | | | 2.2(sd=0.4) | -1.8(sd=0.9) | | -2.0(sd=0.8) | 466(76%) | | 458(75%) |
