# Supplementary material for: Molecular docking studies of 3-bromopyruvate and its derivatives to metabolic regulatory enzymes: Implication in designing of novel anticancer therapeutic strategies
Source: PLoS One. 2017 May 2;12(5):e0176403. doi: 10.1371/journal.pone.0176403 (PMC5413015; doi:10.1371/journal.pone.0176403)
Supplement: S3 Table — (DOCX) [file pone.0176403.s003.docx]

**S3 Table.** Prominent binding site and their residues identification of predicted (SDH) and retrieved (GAPDH, HK 2, LDH, PDH, PGK and IDH 1) protein models by MetaPocket 2.0 server and reported binding site residues from PDB X-ray crystal structures.

| **Protein Name** | **Predicted prominent binding site residues** | **Reported binding site residues** |
| --- | --- | --- |
| **GAPDH** | **Binding site : 1**  Thr^153^, Thr^177^, His^179^, Thr^211^, Ala^232^, Arg^234^, Ala^213^, Ala^209^, Ser^210^, Ser^151^, Cys^152^, Asn^316^, Tyr^314^, Arg^197^, Pro^208^, Gly^212^, Thr^182^, Ile^181^, Thr^237^, Ala^238^, Asn^239^, Glu^317^, Thr^52^, Ser^51^, Arg^13^, Thr^154^, Ile^14^, Asp^50^, Asp^198^, Ile^207^, Gln^185^, Thr^187^, Ala^202^, Leu^203^, Pro^124^, Ala^216^, Ala^183^, Thr^184^, Lys^186^, Lys^215^, Lys^219^ , Met^130^, Ala^150^, Tyr^320^, Leu^195^, Trp^196^, Ala^123^, Ser^122^, Arg^16^, Tyr^49^, Val^188^, Ser^125^, Tyr^45^, Met^46^, Lys^194^, Ser^192^, Ala^126^, Gly^15^, Phe^47^, Gly^12^, Ser^98^, Gly^100^, Thr^103^, Gly^199^, Thr^99^, Glu^97^, Tyr^42^, Gly^193^, Pro^191^, Phe^11^, Ile^121^, Ile^38^, Val^101^, Gly^10^, Asp^35^, Thr^104^, Lys^107^, Phe^37^, Asn^34^, Pro^36^, Asn^9^, Phe^102^, Glu^79^, Arg^80^, Pro^82^, Ile^85^, Asp^81^  **Binding site : 2**  Asn^24^, Ser^288^, Thr^290^, Phe^318^, His^57^, His^53^, Arg^20^, Lys^55^, Phe^56^, Gly^58^, Phe^23^, Asn^70^, Ala^21^, Ser^25^, Asn^322^, Leu^17^, Ser^321^, Gly^54^, Gly^319^  **Binding site : 3**  Glu^138^, Lys^139^, Val^135^, Asn^136^, His^137^, Gly^134^, Pro^269^, Lys^162^, Asp^166^, Glu^223^, His^165^, Ile^221^, Val^220^, Pro^222^, Lys^219^, Pro^129^, Phe^131^, Leu^144^, Met^130^ | Asn^9^, Gly^10^, Phe^11^, Gly^12^, Arg^13^, Ile^14^, Asn^34^, Asp^35^, Pro^36^, Phe^37^, Ile^38^, Arg^80^, Ser^98^, Thr^99^, Gly^100^, Phe^102^, Ser^122^, Ala^123^, Cys^152^, Asp^315^ |
| **HK 2** | **Binding site : 1**  Arg^69^, Val^248^, Glu^249^, Cys^813^, Asp^814^, Met^242^, Phe^67^, Thr^161^, Ile^817^, Lys^162^, Leu^463^, Glu^252^, Thr^812^, Leu^163^, Asp^164^, Arg^470^, His^467^, Val^68^, Val^459^, Ile^818^, Ile^203^, Ala^460^, Asp^815^, Ala^464^, Ser^70^,Arg^769^, Gly^253^, Arg^254^, Gln^466^, Val^456^, Leu^766^, Leu^767^, Phe^768^, Gly^765^, Met^215^, Arg^462^, Thr^71^, Asp^73^, Met^455^, Glu^821^, Leu^474^, Glu^76^, Pro^72^, Gln^471^, Val^825^, Gln^478^, Leu^797^, Gly^250^, Met^247^, Val^822^,Phe^761^, Thr^66^, Asp^465^, Gly^770^, Tyr^461^, Val^207^, Thr^75^  **Binding site : 2**  Thr^88^, Ser^415^, Lys^419^, Asn^89^, Glu^335^, Lys^337^, Thr^232^, Gly^299^, Met^300^, Gly^231^, Thr^336^, Ser^340^, Val^416^, Met^302^, His^420^, Gly^303^, Glu^304^, Gly^332^, Phe^334^, Tyr^301^, Glu^280^, Met^283^, Gly^284^, Arg^307^, Thr^331^,Leu^308^, Ile^281^, Ser^285^,Leu^286^, Lys^295^, Arg^120^, Met^119^, Gly^174^, Gly^87^, Phe^175^, Gly^86^, Lys^173^, Ile^114^, Asp^341^, Glu^116^, Phe^90^, Tyr^112^, Ala^113^, Ile^111^, Leu^85^, Asp^282^, Pro^157^, Thr^172^, Asn^235^, Glu^260^,Gln^291^, Glu^294^, Ser^234^, Gly^262^, Phe^156^, Ser^155^, Asn^287^, Asn^208^, Asn^258^, Gly^233^, Trp^171^, Phe^154^, Asp^209^, Ile^229^, Asn^330^, Arg^333^, Gly^414^, Asp^84^, Ser^449^, Thr^213^, Asp^413^, Pro^115^, Gly^448^, Gly^450^, Lys^418^,Arg^91^, Asp^447^, Ser^445^, Leu^93^, Glu^446^, Met^107^, Asn^109^  **Binding site : 3**  Thr^88^,Gly^231^,Thr^232^, Gly^414^, Ser^415^, Gly^87^, Lys^173^, Asp^209^, Ile^229^, Gly^233^, Ser^234^, Gly^86^, Arg^91^, Asp^413^, Lys^418^, Lys^419^, Arg^444^, Asn^89^, Asp^84^, Gly^448^, Ser^449^, Thr^213^, Leu^85^, Phe^90^, Ser^155^, Phe^156^, Pro^157^, Thr^172^, Asn^208^, Asn^235^, Glu^260^, Gln^291^, Glu^294^, Gly^262^, Asp^447^, Glu^446^, Leu^93^, ASN^109^, Met^107^, Gly^450^, Ser^445^  **Binding site : 4**  Arg^539^, Asn^557^, Glu^894^, Asp^895^, Met^555^, Gly^896^, Asp^532^, Leu^541^, Ser^897^, Gly^898^, Asp^861^, Lys^866^, Gly^534^, Gly^535^, Thr^536^, Asn^537^, Thr^863^, Asp^657^, Ile^677^, Gly^679^, Thr^680^, Gly^862^, Phe^538^, Ser^603^, Lys^621^, Gly^681^, Ser^682^, Asn^683^, Glu^708^, Gly^710^, Gln^739^, Glu^742^, Asn^656^, Thr^620^, Ser^893^, Thr^661^, Ile^559^, Phe^604^, Phe^602^, Pro^605^, Asn^706^ | Glu^46^, Lys^49^, Ala^59^, Ala^60^, Va^l61^, Asp^84^, Gly^87^, Thr^88^, Asn^89^, Thr^153^, Phe^154^, Ser^155^, Phe^156^, Pro^157^, Cys^158^, Thr^172^, Lys^173^, Asn^208^, Asp^209^, Thr^210^, Ile^229^, Gly^131^, Thr^232^, Ser^234^, Asn^235^, Met^242^, Ile^245^, Val^248^, Gly^250^, Glu^260^, Gly^262^, Asp^267^, Gln^291^, Glu^294^, Asp^413^, Gly^414^, Ser^415^, Pro^421^, His^422^, Ala^424^, Lys^425^, Gly^448^, Ser^449^, Ala^458^, Arg^470^, Arg^489^, Asp^532^, Gly^535^, Thr^536^, Ser^603^, Phe^604^ , Pro^605^, Thr^620^, Lys^621^, Asn^656^, Asp^657^, Ile^677^, Gly^679^, Thr^680^, Ser^682^, Asn^683^, Glu^708^, Thr^723^, Glu^724^, Gln^739^, Glu^742^, Asp^861^, Gly^862^, Thr^863^, Gly^896^, Ser^897^ |
| **LDH** | **Binding site : 1**  Tyr^83^, Phe^119^, Ile^120^, Gln^123^, Lys^81^, Asp^82^, Val^53^, Val^51^, Asp^52^, Val^116^, Ala^96^, Val^26^, Gly^97^, Gly^27^, Val^98^, Leu^54^, Thr^95^, Gly^29^, Arg^99^, Ser^137^, Asn^138^, Val^28^, Val^136^, Leu^109^, Lys^57^, Gly^32^, Gln^100^, Val^31^, Gln^30^, Val^140^, Ser^161^, His^193^, Ile^252^, Gly^162^, Arg^106^, Thr^248^, Tyr^247^, Leu^165^, Ile^242^, Ala^238^, Gly^246^, Arg^169^, Asn^249^, Asp^166^, Trp^250^, Ala^251^, Ser^255^, Asn^115^, Asn^164^, Leu^259^  **Binding site : 2**  Val^241^, Lys^245^, Trp^250^, Thr^248^, Asn^249^, Ala^251^, Leu^254^,Arg^169^, Tyr^172^, Ala^168^, Pro^182^, Leu^165^, Ser^255^, Asn^164^, Arg^171^, Ser^183^, His^181^, Trp^188^, Ile^270^, His^271^, Pro^272^, Asp^258^, Arg^269^, Ser^167^, Gly^187^, Lys^42^,Ala^257^, Glu^261^, Ile^252^, Ser^38^, His^186^, Ser^237^, Ala^238^, Met^233^, Val^234^, Leu^173^, Glu^240^, Tyr^239^, Glu^236^, Leu^244^  **Binding site : 3**  Val^198^, Ala^199^, Trp^201^, Asp^311^, Glu^312^, Gln^315^, His^231^, Gly^219^, Lys^228^, Ser^197^, Gln^212^, Lys^309^, Met^218^, Thr^220^, Lys^318^, Pro^216^, Asp^195^, Ser^196^, Val^235^, Lys^232^, Asp^310^, Val^200^, Ser^202^, Asp^221^ | Gly^29^, Gln^30^, Val^31^, Asp^52^, Va^l53^, Leu^54^, Thr^95^, Ala^96^, Gly^97^, Val^98^, Arg^99^, Gln^100^, Arg^106^, Asn^108^, Val^136^, Ser^137^, Asn^138^, Ser^161^, Leu^165^, Arg^169^, His^193^, Ala^238^, Thr^248^, Ile^252^ |
| **SDH** | **Binding site : 1**  Gly^17^, Ser^45^, His^46^, Thr^203^, Gly^52^, Gly^53^, Phe^120^, Arg^287^, Arg^398^, Ala^401^, Gln^51^, His^243^, Leu^253^, Thr^255^, Glu^256^, Ala^50^, Thr^143^, Leu^404^, His^354^, Ser^403^, Ala^49^, Tyr^355^, Thr^47^, Glu^387^, Thr^214^, Leu^407^, Gly^386^, Gly^204^, Gly^18^, Gly^205^, Ser^219^, Ala^19^, Ala^202^, Cys^385^, Gly^20^, Thr^218^, Cys^252^, Thr^333^, Ala^16^, Gly^15^, Leu^40^, Lys^39^, Thr^38^, Ala^216^, Thr^220^, Arg^44^, Val^14^, Val^37^, Asp^222^, Gly^221^, Tyr^166^, Ala^168^, Phe^167^, Met^226^, Ala^183^, Asp^406^, Phe^337^, Gly^329^, Glu^332^, Val^48^, Ile^330^, Asp^141^, Arg^142^, His^145^, Pro^328^, Ser^215^, Gly^207^, Gly^400^, Asn^356^  **Binding site : 2**  Gly^205^, Arg^208^, His^217^, Thr^218^, Ser^219^, Thr^220^, Gly^221^, Leu^518^, Lys^39^, Thr^203^, Asp^222^, Gly^204^, Tyr^206^, Gly^207^, Thr^209^, Tyr^210^, Val^240^, Asn^356^, Ser^215^, His^354^, Glu^387^, His^46^, Val^353^, Tyr^355^, Gln^241^, Ser^45^, Gly^17^, Gly^18^, Thr^47^, Ala^202^, Leu^40^, Gly^15^, Ala^16^, Thr^38^, Ala^216^  **Binding site : 3**  Phe^167^, Ala^168^, Leu^169^, Asp^222^, Arg^229^, Asn^517^, Leu^513^, Asp^170^, Ser^439^, Asn^442^, Gln^516^, Leu^443^, Met^226^, Lys^445^, Val^579^, Tyr^490^, Leu^520^, Leu^493^, Glu^438^, Gly^580^, Leu^171^, Leu^172^, Met^173^, Gly^436^, Leu^446^, Lys^494^, Leu^496^, Gly^491^, Thr^581^, Ala^449^, Ala^230^, Ile^182^, Thr^228^, Ala^435^, Glu^174^, Lys^432^, Ile^431^, Glu^437^, Asn^434^, Met^441^, Gly^582^, Thr^498^, His^495^, Gly^451^, Asp^450^, Phe^448^ |  |
| **PDH** | **Binding site: 1**  Thr^87^, Ala^88^, Gln^14^1, Leu^164^, Tyr^165^, Gly^166^, Glu^194^, Val^138^, Asp^167^, Gly^168^, Ala^169^, Asn^196^, Tyr^198^, Gly^199^, Gln^68^, Gly^139^, Tyr^89^, Phe^94^, Cys^71^, Cys^72^, Ile^137^, Arg^90^, Met^200^, Cys^65^, Ala^91^, Arg^259^, Gly^93^, His^263^, Leu^64^, His^92^, Gly^201^, His^63^, Phe^61^, Ser^264^, Ser^266^, Met^124^, Gly^136^, Asn^195^, Asn^135^  **Binding site: 2**  Lys^118^, Gly^119^, Lys^120^, Pro^330^, Glu^334^, Tyr^337^, His^338^, Val^360^, Pro^331^, Leu^332^, Leu^335^, Cys^116^, Ala^117^, Lys^113^, Gly^115^, Glu^329^, Glu^333^, Gly^121^, His^125^, Tyr^127^, Gly^336^, Ile^339^, Val^348^, Arg^349^, Phe^357^, Gly^350^, Ala^351^, Tyr^340^, Ser^341^, Ser^361^, Ser^342^  **Binding site: 3**  Ala^326^, Pro^328^, Arg^112^, Asp^327^, Glu^329^, Thr^110^, Lys^56^, Ile^57^, Arg^59^, Ile^58^, Leu^109^, Gly^111^, Gly^119^, Lys^120^, Gly^121^, Gly^122^, Phe^61^, Ser^123^, Met^124^, His^125^, Glu^108^, Gly^60^, Lys^118^, Pro^330^, Cys^62^, Leu^335^, Ala^117^, Glu^334^, Pro^331^, Leu^332^, Glu^333^, Phe^35^ | Tyr^89^, Arg^90^, Gly^136^, Val^138^, Lys^153^, Gly^166^, Asp^167^, Gly^168^, Ala^169^, Trp^185^, Leu^187^, Asn^196^, Tyr^198^, Gly^199^, His^263^ |
| **PGK** | **Binding site : 1**  Lys^30^, Asn^31^, Glu^294^, Trp^345^, Asn^295^, Asp^293^, Glu^344^, Met^29^, Val^342^, Asn^32^, Phe^292^, Thr^35^, Gln^33^, Asp^74^, Lys^75^, Pro^28^, Asp^375^, Gly^341^, Phe^343^, Gly^313^, Leu^314^, Asp^315^, Val^340^, Gly^238^, Phe^242^, Cys^316^, Pro^80^, Pro^339^, Pro^73^, Gly^239^, Leu^257^, Met^312^, Ser^256^, Thr^378^, Ser^77^, Tyr^76^, Ala^215^, Thr^255^, Glu^79^, Val^27^, Leu^64^, Gly^214^, Gly^338^, Met^72^, Thr^376^, Gly^213^, Gly^374^, Asn^26^, Gly^65^, Lys^216^, Arg^66^, Arg^39^, Asn^337^, Gly^373^, Lys^220^, Gly^396^, Val^217^, Gly^397^, His^63^, Arg^123^, Asp^24^, Gly^167^, Thr^168^, His^170^, Arg^171^, Asp^219^, Asp^68^, Glu^129^, His^173^, Lys^131^, Ala^132^, Gly^130^, Glu^128^, Lys^382^, Phe^286^, Ala^241^, Pro^283^, Tyr^324^, Leu^223^, Glu^401^, Ala^218^, Gln^222^, Lys^406^, Met^240^, Leu^212^, Asp259, Phe258,  **Binding site : 2**  Lys^41^, Pro^45^, Lys^192^, Ala^42^, Leu^189^, Ser^46^, Asp^164^, Phe^188^, Glu^386^, Val^389^, Ser^390^, His^391^, Val^392^, Asp^387^, Gln^38^, Asn^36^, Ala^377^, Ala^381^, Thr^394^, Glu^193^, Tyr^196^, Asn^37^, Arg^39^  **Binding site : 3**  Ala^215^, Phe^292^, Val^342^, Glu^344^, Gly^214^, Gly^238^, Gly^239^, Leu^314^, Pro^339^, Val^340^, Gly^341^, Asp^375^, Phe^343^, Gly^313^, Asp^315^, Thr^255^, Ser^256^, Leu^257^, Met^312^, Phe^242^, Pro^283^, Phe^286^, Cys^316^, Ala^241^, Tyr^324^ | Asp^24^, Asn^26^, Arg^39^, His^63^, Arg^66^, Arg^123^, Gly^167^, Thr^168^, Arg^171^, Gly^214^, Ala^215^, Lys^216^, Asp^219^, Lys^220^, Gly^238^, Gly^239^, Phe^242^, Leu^257^, Gly^313^, Leu^314^, Asn^337^, Pro^339^, Gly^341^, Val^342^, Glu^344^, Gly^373^, Gly^374^, Asp^375^, Thr^376^, Gly^396^, Gly^397^ |
| **IDH 1** | **Binding site : 1**  Arg^100^, Tyr^272^, His^132^, His^133^, Asn^271^, Asp^273^, Gly^274^, Asp^275^, Ala^134^, Tyr^135^,Val^107^, Arg^109^, Gln^277^, Val^276^, Ser^293^, Glu^306^, Ala^307^, Ala^308^, Lys^72^, Cys^73^, Ala^74^, Thr^75^, Asn^96^, His^309^, Ser^94^, Thr^77^, Gly^310^, Thr^311^, Gln^283^, Leu^288^, Thr^19^, Trp^23^, Ser^332^, Ala^335^, Ile^22^, Phe^108^, Ile^130^, Asp^279^, Glu^110^, Met^291^, Ala^282^, Val^281^, Trp^267^, Gly^136^, Gly^97^, Asn^101^, Asp^137^, ^Ile76,^ Tyr^139^, Thr^98^,  **Binding site : 2**  Ala^111^, Tyr^285^, Gly^286^, Ser^287^, Met^291^, Val^281^, Ala^282^, Pro^118^, Met^290^, Leu^120^, Arg^119^, Ile^112^, Trp^124^, Ile^117^, Ile^113^, Cys^379^, Leu^376^, Asp^279^, Ile^130^, Cys^114^, Ile^128^, Arg^109^, Glu^110^, Pro^127^, Lys^126^, Trp^267^, Ala^258^, Met^259^, Val^255^, Phe^265^, Met^254^, Ile^251^, Ser^278^, Ser^280^, His^132^, Asp^273^, Gly^274^, Asp^275^, Cys^269^, Asn^271^, Leu^288^, Gln^283^, Gly^284^, Gly^289^, Gln^277^, Val^276^, Tyr^272^, Asn^116^, Lys^115^, Val^121^, Ser^210^, Ala^256^, Asp^252^,  **Binding site : 3**  Phe^108^, Glu^110^, Ile^129^, Thr^292^, Val^294^, Arg^338^, Lys^203^, Ser^293^, Met^199^, Val^296^, Val^303^, Ser^202^, Ala^341^, Glu^360^, His^342^, Lys^345^, Gln^198^, Gly^300^, Ser^195^, Thr^302^, Thr^337^, Ala^356^, Asn^357^, Ala^353^, Cys^297^, Phe^354^, Arg^109^, Ile^112^, Ile^113^, Cys^114^, Pro^127^, Phe^33^4, Ile^364^, Ala^111^, Ile^128^, Ala^305^, Gly^339^, Ile^130^, Leu^295^, Lys^115^, Glu^368^, Arg^119^, Val^125^, Glu^361^, Lys^126^, Trp^205^, Glu^365^, Trp^124^, Ala^358^, Asp^299^, Gly^264^, Leu^346^, Pro^298^, Gly^204^, Leu^201^, Ala^344^, Glu^306^, Ala^307^, Ala^335^, Asn^348^, Asn^349^, Lys^350^ | Lys^72^, Ala^74^, Thr^75^, Ile^76^, Thr^77^, Arg^82^, Asn^96^, Trp^128^, Ile^130^, Met^254^, Ala^258^, Trp^267^, Gln^277^, Asp^279^, Val^281^, Ala^282^, Gly^289^, Met^291^, His^309^, Gly^310^, Thr^311^, Val^312^, Thr^313^, Arg^314^, His^315^, Asn^328^ |
